# Endoscopic Evaluation and Management of Diversion Pouchitis and Strictures: In Comparison With Strictures in Nondiverted Pouches

**DOI:** 10.1016/j.gastha.2026.101103

**Published:** 2026-08-21

**Authors:** Yuanyuan Ge, Ellen L. Scherl, Dana J. Lukin, Joseph Picoraro, Marco Bertucci Zoccali, Le-Chu Su, Usama Ahmed Ali, Debbie Bakes, Ravi P. Kiran, James M. Church, Bo Shen

**Affiliations:** 1 Center of Inflammatory Bowel Disease, Columbia University Irving Medical Center/New York Presbyterian Hospital, New York, New York; 2 Jill Roberts Center for Inflammatory Bowel Disease, Gastroenterology and Hepatology, Department of Medicine, New York Presbyterian/Weill Cornell Medical Center, New York, New York; 3 Division of Pediatric Gastroenterology, Columbia University Irving Medical Center/Morgan Stanley Hospital of New York Presbyterian Hospital, New York, New York

**Keywords:** Stricturotomy, Ileal Pouch, Stricture, Diverted Pouchitis, Ulcerative Colitis

## Abstract

**Background and Aims:**

Postoperative complications after ileal pouch-anal anastomosis are common and may lead to pouch failure requiring long-standing fecal diversion. Patients with diverted pouches can develop diversion pouchitis and strictures; however, the endoscopic features and management of these conditions remain poorly characterized.

**Methods:**

This historical case-controlled study included 54 consecutive inflammatory bowel disease patients with ileal pouch-anal anastomosis who underwent endoscopic evaluation and stricturotomy for distal pouch or anastomotic strictures from January 2020 to February 2026. Twelve consecutive patients with diverted pouch strictures were compared with 42 nondiverted controls. Outcomes included pouchitis frequency, stricture characteristics, technical success, endoscopic reintervention, surgery-free survival, and adverse events. Kaplan-Meier and Cox regression analyses were used for survival end points.

**Results:**

Among diverted patients, 75% (N = 9) had concurrent diversion pouchitis. Strictures in diverted pouches were uniformly severe (100% vs 7.1%, *P* < .001) and longer (median 1.5 vs 1.0 cm, *P* = .002), with higher Pouchitis Disease Activity Index endoscopic subscores (median 2.0 vs 1.0, *P* < .001). Overall technical success was 98.4% (121/123 procedures), comparable between groups (95.2% vs 100%, *P* = .115). Surgical intervention was required in 3 (25%) diverted vs 9 (21%) nondiverted patients (*P* > .99); endoscopic reintervention occurred in 6 (50%) vs 18(43%) cases, respectively (*P* = .7). Surgery-free survival at 12 and 24 months was 88.9% and 47.4% in diverted vs 86.2% and 86.2% in nondiverted patients (log-rank *P* = .433; hazard ratio 1.68, 95% confidence interval 0.45–6.32). Adverse events occurred in 1 diverted (8.33%; abscess) vs 1 nondiverted patient (2.38%; ileus), with no perforation, bleeding, or mortality.

**Conclusion:**

Most diverted pouch patients had concurrent diversion pouchitis with more severe and longer strictures. Despite these differences, endoscopic stricturotomy was feasible and safe with comparable outcomes to nondiverted pouch strictures.

## Introduction

Restorative proctocolectomy with ileal pouch-anal anastomosis (IPAA) is the preferred surgical treatment for patients with medically refractory ulcerative colitis (UC), colitis-associated neoplasia, and familial adenomatous polyposis.^1^^,^^2^ Although IPAA substantially improves health-related quality of life by preserving the natural route of defecation, the construction and maintenance of the ileal pouch are associated with various structural, inflammatory, and functional complications.^3^^,^^4^ Postoperative complications occur in a substantial proportion of patients, with stricture formation reported in 5%–38% of IPAA patients.^1^^,^^5^ Severe or refractory complications can lead to pouch failure, necessitating the construction of a diverting ileostomy and long-term fecal diversion.^1^^,^^6^

Patients with diverted pouches can develop diversion pouchitis, a diffuse inflammatory condition resulting from the exclusion of intestinal continuity.^4^ The long-term absence of luminal flow can also result in stricture formation in the diverted pouch. Diversion-associated strictures may further prevent ileostomy closure, contribute to pouch dysfunction, and increase the risk for pouch excision.^6^^,^^7^ The management of diversion-associated strictures in patients with underlying inflammatory bowel disease (IBD) can be challenging, as conventional approaches such as bougie dilation or endoscopic balloon dilation (EBD) may not be safe or effective for severe fibrotic strictures with friable tissues.^8^^,^^9^ Endoscopic stricturotomy with an insulation-tipped knife or needle knife has emerged as an effective and safe modality for treating fibrotic pouch strictures^5^^,^^10^^,^^11^; however, data on the endoscopic features and treatment outcomes of strictures in diverted pouches are scant, with only case reports available.^12^^,^^13^ We therefore conducted a case-controlled study to characterize the endoscopic features of diversion-associated inflammation and strictures, and to evaluate the efficacy and safety of endoscopic stricturotomy in this population, compared to patients with nondiverted pouch strictures.

## Patients and Methods

### Study Design and Data Source

This historical case-controlled study was approved by the Columbia University Irving Medical Center’s Institutional Review Board (IRB#AAAN3966). Consecutive IBD patients with IPAA surgeries who underwent endoscopic evaluation and stricturotomy for distal pouch or pouch-anal anastomotic strictures at the Center for Inflammatory Bowel Disease from January 2020 to February 2026 were studied. Patients’ demographic, clinical, endoscopic, and outcome data were collected from the prospectively maintained Pouch Registry, a single-center registry that consecutively enrolled all patients with an ileal pouch undergoing endoscopic evaluation at our center, regardless of disease severity or procedure type.

### Inclusion Criteria and Exclusion Criteria

Inclusion criteria were patients (1) aged ≥18 years at the time of the index endoscopic treatment, (2) with underlying diagnosis of IBD (UC, Crohn’s disease [CD]), (3) with a history of IPAA with J or S pouch, (4) with documented stricture at the distal pouch or pouch-anal anastomosis (outlet/anastomotic site), (5) with endoscopic stricturotomy alone for the distal pouch/anastomotic stricture, or (6) being classified into the diverted group (diverting ileostomy present at the time of index endoscopic stricturotomy) or the nondiverted group (no diverting ileostomy at the time of index endoscopic stricturotomy). Exclusion criteria were patients with (1) Kock, W, or H pouches; (2) colectomy and IPAA for familial adenomatous polyposis or other non-IBD indications; or (3) concomitant strictures at other pouch locations (inlet, afferent limb, pouch body, or loop ileostomy closure site) or (4) endoscopic treatment with balloon dilation only, strictureplasty only, or combination therapies (eg, balloon dilation + stricturotomy, stricturotomy + strictureplasty).

### Demographic and Clinical Variables

Demographic and clinical variables included age at the time of procedure, sex, race/ethnicity, body mass index, smoking status, the American Society of Anesthesiologists physical status classification, and first-degree family history of IBD. Clinical data included the present diagnosis, duration of IBD, duration of IBD before colectomy, indication for colectomy, concurrent use of chronic nonaspirin nonsteroidal anti-inflammatory drug, preoperative biologics or small-molecule agents, chronic antibioticS (>4 weeks per course at the time of pouchoscopy), and extraintestinal manifestations.

Surgical variables included age at pouch creation, duration of pouch, pouch type (J or S), anastomosis type (stapled or hand-sewn), and mucosectomy status. Postoperative use of immunomodulators, biologics, and small molecule agents was recorded, along with the indication for each (pouch disorders vs autoimmune disorders or organ transplantation).

### Endoscopic Assessment and Treatment of Strictures

The 6-point Pouchitis Disease Activity Index (PDAI) endoscopic subscore was evaluated during each pouchoscopy. Pouchitis, including diversion pouchitis, was diagnosed by the treating endoscopist based on clinical and endoscopic assessment documented in the medical chart, endoscopic reports, and saved photographs. Underlying pouch disorders were classified as follows: (1) chronic pouchitis, (2) cuffitis, and (3) surgical complications (conditions attributed to surgical techniques, including pouch sinuses, fistulae, pouch ischemia, and pouch prolapse). The diagnosis of stricture was made based on endoscopy, regardless of the presence or absence of stricture-related symptoms. Stricture severity was graded as mild, moderate, or severe/pinhole based on the degree of resistance to or ability of endoscope passage, according to our previously published classification.^14^ Stricture length (cm) was recorded.

### Endoscopic Treatment Protocol

Outpatient or inpatient pouchoscopy was performed using a gastroscope. Endoscopic stricturotomy was performed using an insulated-tip (Olympus, Tokyo, Japan) and/or needle knife (Boston Scientific, Marlborough, MA) for fibrotic strictures. All stricturotomy procedures were performed by a single endoscopist with 30 years of experience in IBD and therapeutic endoscopy. The choice of electrosurgical knife was at the discretion of the endoscopist based on the stricture characteristics. Hemostatic agents (hypertonic glucose, hemostatic gel, or both) were used as needed to prevent or control bleeding. The cumulative number of pouchoscopy with stricture therapy was recorded.

### Outcome Measurement

The primary outcome was the immediate technical success of endoscopic stricturotomy. Technical success was defined as achieving endoscopic passage through the stricture (passage of a gastroscope with or without resistance for nontraversable strictures after the endoscopic therapy; or passage of the scope without resistance of traversable-but-resistant strictures after endoscopic therapy).^15^ Secondary outcomes were the frequency of diversion pouchitis, endoscopic characteristics of strictures, endoscopic reintervention (defined as need for repeat endoscopic treatment after initial success), requirement of surgical intervention for the stricture (ie, surgical dilation, excision of the pouch, or pouch redo), and diversion-associated or procedure-related adverse events (bacteremia, sepsis, abscess, bleeding, perforation, and mortality). Procedure-related adverse events were assessed within a 7-day surveillance window after each procedure. Subsequent surgeries were counted as adverse events only if they occurred within this window.

### Statistical Analysis

Descriptive statistics were computed for all variables. Continuous variables were expressed as mean ± standard deviation or median with interquartile range (IQR) as appropriate. Categorical variables were presented as frequencies and percentages. Comparisons between diverted and nondiverted groups were performed using the Welch two-sample *t*-test or Wilcoxon rank-sum test for continuous variables and Chi-square tests or Fisher exact tests (when expected cell counts <5) for categorical variables. Major complication rates were calculated per patient and per endoscopic procedure. Kaplan-Meier analysis was used to estimate surgery-free survival and endoscopic re–intervention-free survival, with survival rates reported at 12 and 24 months. Log-rank tests were used to compare survival curves between diverted and nondiverted groups. Cox proportional hazards regression was performed to estimate hazard ratios (HRs) with 95% confidence intervals (CIs). Statistical analyses were performed using R (version 4.3.0; R Foundation for Statistical Computing, Vienna, Austria). A two-sided *P* value <.05 was considered statistically significant.

## Results

A total of 54 patients with IPAA for IBD who underwent endoscopic stricturotomy for distal pouch or anastomotic strictures were included: 12 consecutive patients with diverted pouches and 42 consecutive patients with nondiverted pouches. Among the 12 diverted patients, 11 had completed all stages of restorative proctocolectomy with IPAA and were subsequently diverted for pouch-related complications; the remaining patient had not completed the final stage due to a concurrent enterocutaneous fistula and remained diverted for approximately 2 years, during which a pouch stricture and diversion pouchitis developed, and was included in the diverted group. The median duration of fecal diversion was 2.2 (IQR 1.4, 6.0) years. Among the 11 rediverted patients, the predominant indication was pouch-related fistulae (N = 9 [82%]), including complex perianal fistula (N = 3), pouch-vesical fistula (N = 2), concurrent pouch-vesical and perianal fistula (N = 2), pouch-urethral fistula (N = 1), and pouch-prostate fistula (N = 1); the remaining 2 (18%) were diverted for chronic pouchitis with pouch dysfunction.

Of the entire cohort, 41 (76%) patients had underlying UC and 13 (24%) had CD as the latest diagnosis, based on a confirmed preoperative or immediate postoperative diagnosis of CD or CD of the pouch. The two groups were well matched with respect to baseline demographic and clinical characteristics, including age, sex, ethnicity, body mass index, smoking history, and the American Society of Anesthesiologists classification (Table 1). However, patients in the diverted group had a significantly shorter median duration of IBD before colectomy (2 [IQR 1, 2] vs 6 [IQR 1, 14] years, *P* = .035). A numerically higher proportion of diverted patients had CD as the present diagnosis (N = 5 [42%] vs N = 8 [19%], *P* = .134).

**Table 1:** Clinical Characteristics of Patients With and Without Fecal Diversion

Variable	Overall N = 54^a^	With diversion N = 12^a^	Without diversion N = 42^a^	*P* value^b^
Age, y	47 ± 16	45 ± 18	48 ± 15	.564
Female	36 (67%)	8 (67%)	28 (67%)	1.000
Ethnicity				.551
Caucasian	43 (80%)	11 (92%)	32 (76%)	
African American	1 (1.9%)	0 (0%)	1 (2.4%)	
Asian/other	10 (19%)	1 (8.3%)	9 (21%)	
Median body mass index, kg/m^2^	21.6 (19.9, 26.6)	23.8 (19.9, 26.8)	21.5 (19.7, 26.6)	.819
Smoking history				.249
Never	48 (89%)	10 (83%)	38 (90%)	
Ex-smoker	4 (7.4%)	2 (17%)	2 (4.8%)	
Current	2 (3.7%)	0 (0%)	2 (4.8%)	
ASA classification				.538
I	1 (1.9%)	0 (0%)	1 (2.4%)	
II	44 (81%)	9 (75%)	35 (83%)	
III	9 (17%)	3 (25%)	6 (14%)	
IV	0 (0%)	0 (0%)	0 (0%)	
Family history of IBD	2 (4.0%)	1 (8.3%)	1 (2.6%)	.426
Median duration of IBD before colectomy, y	3 (1, 9)	2 (1, 2)	6 (1, 14)	**.035**
Median duration of IBD, y	18 (10, 32)	21 (9, 35)	18 (10, 30)	.918
Present diagnosis				.134
Ulcerative colitis	41 (76%)	7 (58%)	34 (81%)	
Crohn's disease	13 (24%)	5 (42%)	8 (19%)	
Concurrent use of chronic NSAID	4 (7.5%)	0 (0%)	4 (9.8%)	.563
Preoperative medication				1.000
None	24 (51%)	6 (50%)	18 (51%)	
Biologics	23 (49%)	6 (50%)	17 (49%)	
Small molecules	0 (0%)	0 (0%)	0 (0%)	
Primary sclerosing cholangitis	1 (1.9%)	0 (0%)	1 (2.4%)	1.000
Arthralgia/arthropathy	11 (20%)	1 (8.3%)	10 (24%)	.421
Cutaneous lesions	1 (1.9%)	0 (0%)	1 (2.4%)	1.000
Ocular lesions	0 (0%)	0 (0%)	0 (0%)	1.000
Indication for colectomy				.306
Refractory disease	49 (91%)	10 (83%)	39 (93%)	
Dysplasia or cancer	5 (9.3%)	2 (17%)	3 (7.1%)	

Bold values indicate statistical significance (*P* <.05).

ASA, American Society of Anesthesiologists; NSAID, nonsteroidal anti-inflammatory drug; SD, standard deviation.

a Mean ± SD; n (%); median (Q1, Q3).

b Welch two-sample *t*-test; Fisher exact test; Wilcoxon rank-sum test.

Pouch and procedural characteristics are detailed in Table 2. Pouch type, anastomosis type, and duration of the pouch were comparable between groups. Mucosectomy was performed significantly less frequently in diverted patients (N = 1 [8.3%] vs N = 20 [48%], *P* = .018). Postoperative use of small molecule agents differed significantly between groups (*P* = .046), driven by upadacitinib use in 2 diverted patients (17%), while a trend toward higher postoperative biologics use was observed in the diverted group (N = 9 [75%] vs N = 20 [48%], *P* = .077).

**Table 2:** Procedure Characteristics in Patients With and Without Fecal Diversion

Variable	Overall N = 54^a^	With diversion N = 12^a^	Without diversion N = 42^a^	*P* value^b^
Mean age at pouch creation, y	28 (19, 41)	24 (17, 43)	29 (21, 37)	.318
Median duration of pouch, y	14 (8, 29)	13 (8, 34)	14 (8, 27)	.755
Pouch type				.306
J-pouch	49 (91%)	10 (83%)	39 (93%)	
S-pouch	5 (9.3%)	2 (17%)	3 (7.1%)	
Anastomosis type				.328
Stapled	47 (87%)	12 (100%)	35 (83%)	
Handsewn	7 (13%)	0 (0%)	7 (17%)	
Mucosectomy	21 (39%)	1 (8.3%)	20 (48%)	**.018**
Chronic antibiotic use for pouch	36 (67%)	6 (50%)	30 (71%)	.184
Postoperative use of immunomodulators				1.000
None	51 (94%)	12 (100%)	39 (93%)	
For autoimmune disorders or organ transplantation	1 (1.9%)	0 (0%)	1 (2.4%)	
For pouch disorders	2 (3.7%)	0 (0%)	2 (4.8%)	
Postoperative use of biologics				.077
None	25 (46%)	3 (25%)	22 (52%)	
For autoimmune disorders or organ transplantation	8 (15%)	4 (33%)	4 (9.5%)	
For pouch disorders	21 (39%)	5 (42%)	16 (38%)	
Postoperative use of small molecule agents				**.046**
None	51 (94%)	10 (83%)	41 (98%)	
Tofacitinib	1 (1.9%)	0 (0%)	1 (2.4%)	
Upadacitinib	2 (3.7%)	2 (17%)	0 (0%)	
Underlying pouch disorder with and without current diversion				.148
None	6 (11%)	1 (8.3%)	8 (19%)	
Chronic pouchitis	15 (28%)	9 (75%)	15 (36%)	
Cuffitis	5 (9.3%)	0 (0%)	5 (12%)	
Surgical complications	15 (28%)	2 (17%)	14 (33%)	
Median Pouchitis Disease Activity Index endoscopic subscore	1.00 (0.00, 2.00)	2.00 (2.00, 3.00)	1.00 (0.00, 2.00)	**.000**
Stricture degree				**.000**
Mild	19 (35%)	0 (0%)	19 (45%)	
Moderate	20 (37%)	0 (0%)	20 (48%)	
Severe/pinhole	15 (28%)	12 (100%)	3 (7.1%)	
Median stricture length, cm	1.00 (1.00, 1.50)	1.50 (1.50, 2.50)	1.00 (1.00, 1.50)	**.002**
Median number of pouchoscopies with stricture therapy	1.00 (1.00, 3.00)	2.00 (1.00, 3.00)	1.00 (1.00, 2.00)	.300
Knife type				.161
Insulated tip (IT)	38 (70%)	6 (50%)	32 (76%)	
Needle knife (NK)	6 (11%)	2 (17%)	4 (9.5%)	
IT and NK	10 (19%)	4 (33%)	6 (14%)	
Preventing bleeding				**.007**
None	42 (78%)	6 (50%)	36 (86%)	
Hypertonic glucose	5 (9.3%)	1 (8.3%)	4 (9.5%)	
Hemostatic gel	6 (11%)	4 (33%)	2 (4.8%)	
Hypertonic glucose and hemostatic gel	1 (1.9%)	1 (8.3%)	0 (0%)	
Surgical intervention required after index endoscopy	12 (22%)	3 (25%)	9 (21%)	1.000
Surgical procedure type				1.000
Surgical dilation	6 (50%)	1 (33%)	5 (56%)	
Pouch excision	6 (50%)	2 (67%)	4 (44%)	
Pouch redo	0 (0%)	0 (0%)	0 (0%)	
Endoscopic reintervention required	24 (44%)	6 (50%)	18 (43%)	.748
Median follow-up, mo	24 (8, 52)	15 (6, 38)	28 (8, 52)	.298
Median number of clinic visits	4.0 (2.0, 8.0)	6.0 (2.5, 9.0)	3.5 (2.0, 6.0)	.270
Postprocedure medication				.284
None	6 (11.1%)	0 (0%)	6 (14.3%)	
Mesalamines	5 (9.3%)	0 (0%)	5 (11.9%)	
Corticosteroids	2 (3.7%)	1 (8.3%)	1 (2.4%)	
Biologics	24 (44.4%)	7 (58.3%)	17 (40.5%)	
Immunomodulators	0 (0%)	0 (0%)	0 (0%)	
Antibiotics	15 (27.8%)	3 (25.0%)	12 (28.6%)	
Small molecules	2 (3.7%)	1 (8.3%)	1 (2.4%)	

Bold values indicate statistical significance (*P* <.05).

a Median (Q1, Q3); n (%).

b Wilcoxon rank-sum test; Fisher exact test; Wilcoxon rank-sum exact test.

### Characterization of Diversion-Associated Inflammation and Stricture

Among 12 diverted patients, 9 (75%) had concurrent diversion pouchitis. The distribution of underlying pouch disorders did not differ significantly between groups (*P* = .148). The median PDAI endoscopic subscore was significantly higher in the diverted group (2.0 [IQR 2.0, 3.0] vs 1.0 [IQR 0.0, 2.0], *P* < .001), consistent with the high prevalence of diversion pouchitis.

Strictures in diverted pouches were uniformly severe: all 12 patients (100%) presented with severe or pinhole strictures, compared with only 3 (7.1%) patients in the nondiverted group (*P* < .001), where mild (N = 19 [45%]) and moderate (N = 20 [48%]) strictures were predominant. The median stricture length was significantly greater in the diverted group (1.5 [IQR 1.5, 2.5] vs 1.0 [IQR 1.0, 1.5] cm, *P* = .002). Representative pictures of diverted and nondiverted strictures are shown in Figure 1.

**Figure 1:**
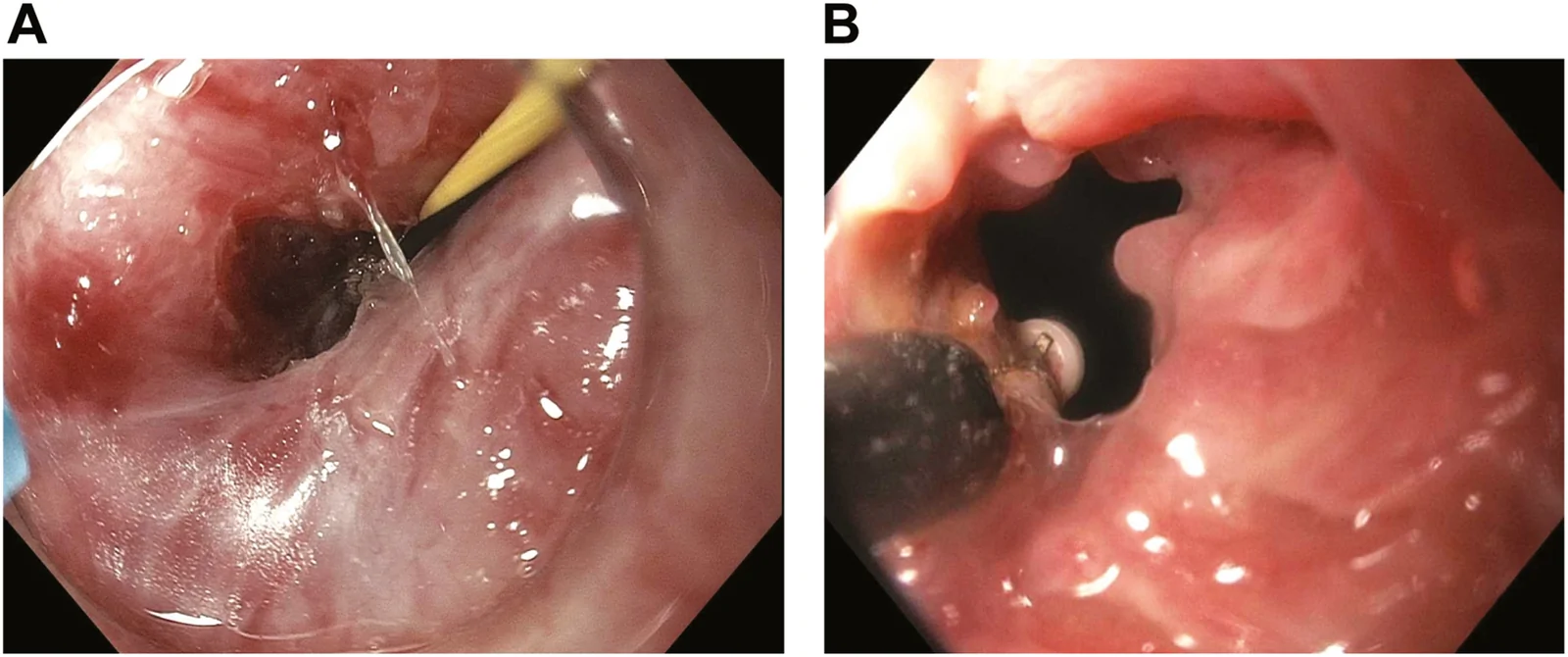
Representative endoscopic pictures of diverted and nondiverted pouch strictures. (A) A severe, pinhole diverted-pouch stricture with friable, inflamed, and edematous mucosa characteristic of concurrent diversion pouchitis. (B) A non–diverted-pouch stricture with a wider, more distensible lumen and comparatively less mucosal inflammation.

### Efficacy of Endoscopic Stricturotomy

A total of 123 endoscopic stricturotomy procedures were performed: 42 in 12 diverted patients and 81 in 42 nondiverted patients. All index and repeat interventions were endoscopic stricturotomy. The overall technical success rate was 121/123 (98.4%), comparable between diverted and nondiverted patients (40/42 [95.2%] vs 81/81 [100.0%], Fisher exact *P* = .115). The median number of pouchoscopies with stricture therapy was numerically higher in the diverted group, although this did not reach statistical significance (2.0 [IQR 1.0, 3.0] vs 1.0 [IQR 1.0, 2.0], *P* = .300). The insulation tipped knife was the most commonly used instrument overall (N = 38 [70%]), and knife type did not differ between groups (*P* = .2). The use of hemostatic agents for bleeding prevention was significantly more frequent in the diverted group (N = 6 [50%] vs N = 6 [14%], *P* = .007).

Surgical intervention after initial endoscopic treatment was required in 3 (25%) diverted and 9 (21%) nondiverted patients (*P* > .99). In the diverted group, 1 patient underwent surgical dilation for anastomotic stricture and 2 had pouch excision (1 for pouch-prostate fistula, 1 for pouch dysfunction after stoma closure). In the nondiverted group, 5 underwent surgical dilation and 4 had pouch excision (2 for pouch-vesical fistula, 1 for recurrent perianal fistula, 1 for pouch dysfunction). Endoscopic reintervention was required in 6 (50%) diverted and 18 (43%) nondiverted patients (*P* = .748). Kaplan-Meier analysis demonstrated a surgery-free survival rate at 12 and 24 months of 88.9% and 47.4% in the diverted group vs 86.2% and 86.2% in the nondiverted group (log-rank *P* = .433; HR 1.68, 95% CI 0.45–6.32; Figure 2). Endoscopic re–intervention-free survival at 12 and 24 months was 57.1% and 28.6% in the diverted group vs 66.5% and 50.4% in the nondiverted group (log-rank *P* = .118; HR 2.07, 95% CI 0.81–5.27; Figure 3). The median follow-up was 15 (IQR 6, 38) vs 28 (IQR 8, 52) months (*P* = .3). Postprocedural medication use was comparable between groups (*P* = .4). Among the 12 diverted patients, 4 received therapy directed specifically at diversion pouchitis: topical corticosteroids in 1, oral antibiotics in 2, and an antibiotic combined with a short-chain fatty acid enema in 1 patient. The remaining biologic and small-molecule agents were directed at the underlying IBD disease. During follow-up, only 2 (17%) achieved successful stoma closure during follow-up; 1 additional patient underwent stoma closure but ultimately required pouch excision with conversion to a Kock pouch 5 months later due to pouch dysfunction.

**Figure 2:**
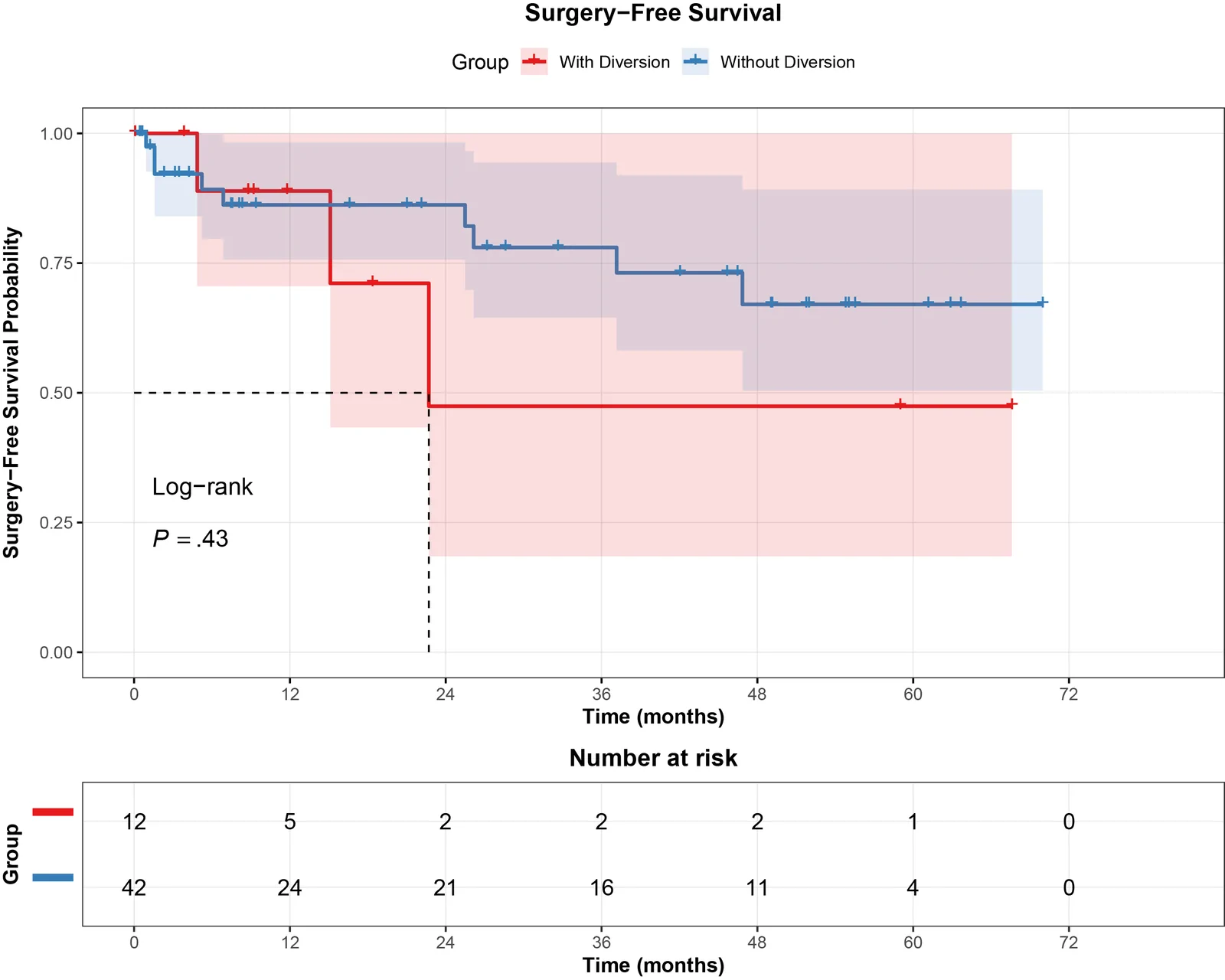
Surgery-free survival curves.

**Figure 3:**
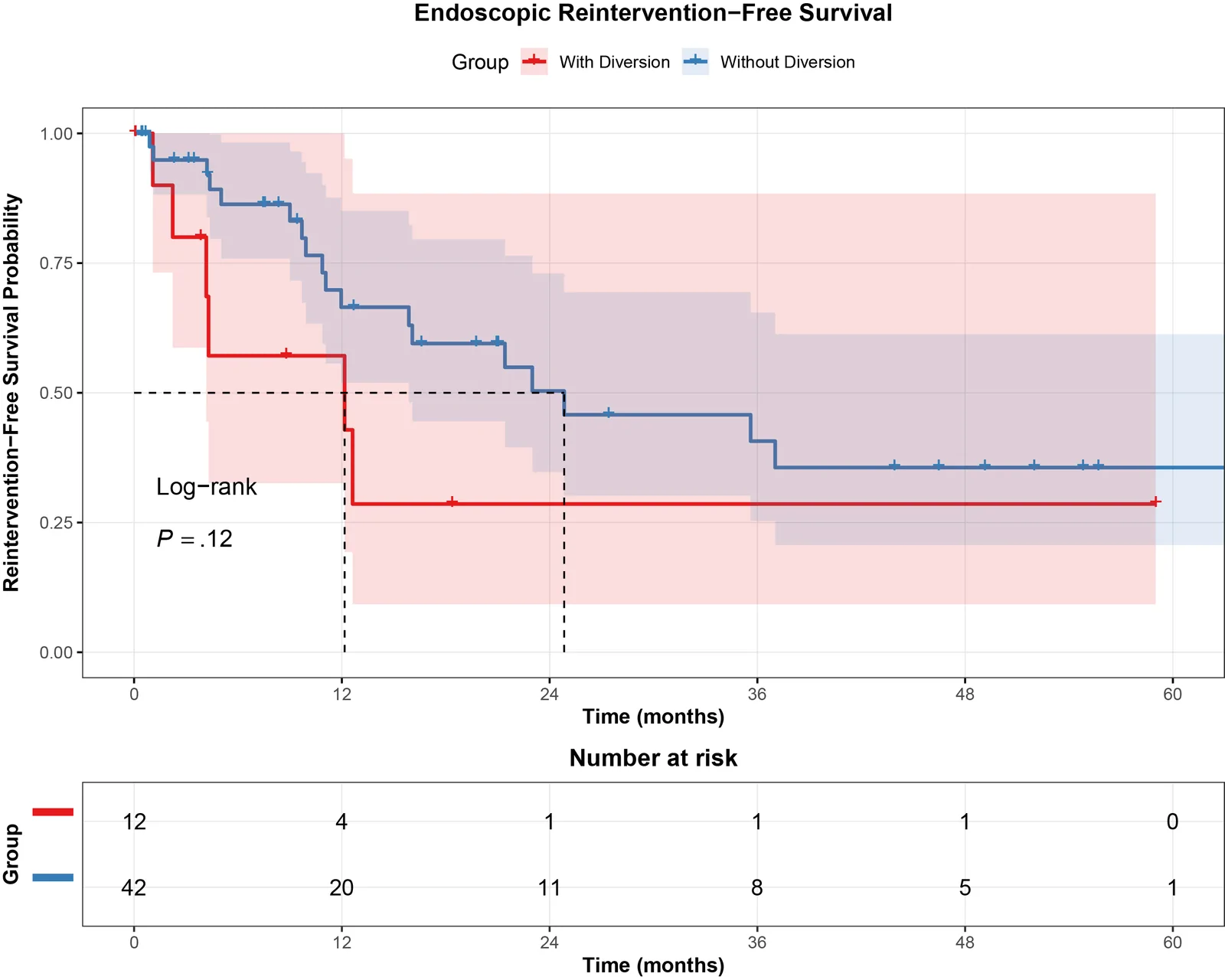
Endoscopic reintervention-free survival curves.

### Adverse Events of Diversion Pouchitis, Stricture, and Endoscopic Procedure

Two adverse events met the predefined criteria for procedure-related adverse events within the 7-day surveillance window (Table 3). In the diverted group, 1 patient developed a postprocedure pelvic abscess with bacteremia (on biologic therapy). In the nondiverted group, the single adverse event was a postprocedure ileus. Two additional infectious events were judged highly relevant to diversion status but did not meet the adverse-event criteria, as they were not attributable to a procedure performed at our institution within the surveillance window: 1 patient, though having undergone successful endoscopic treatment without any adverse events at our institution, was thought to have diverted pouch-associated bacteremia and liver abscess after a failed endoscopic treatment for pouch strictures at an outside hospital prior to referral. Another patient with a preexisting pouch-prostate fistula experienced a urinary tract infection after endoscopic treatment (not on biologic or small-molecule therapy). There were no cases of procedure-associated bleeding, perforation, or mortality in either group across all 123 procedures.

**Table 3:** Complications of Endoscopic Therapy per Patient-Based and per Procedure-Based (Total Events = 2)

Adverse event	With diversion			Without diversion		
	Number of adverse events	Number of patients treated (N = 12)	Number of endoscopy performed (N = 42)	Number of adverse events	Number of patients treated (N = 42)	Number of endoscopy performed (N = 81)
Postprocedure bacteremia or abscess	1	8.33%	2.38%	0	0%	0%
Postprocedure ileus	0	0%	0%	1	2.38%	1.23%
Procedure-associated bleeding	0	0%	0%	0	0%	0%
Procedure-associated perforation	0	0%	0%	0	0%	0%
Procedure-associated mortality	0	0%	0%	0	0%	0%

## Discussion

To our knowledge, this is the first case-controlled study to characterize the endoscopic features of diversion-associated inflammation and strictures in ileal pouches and to evaluate the efficacy and safety of endoscopic stricturotomy in this population. Our findings demonstrate that diverted pouch patients had a high prevalence of concurrent diversion pouchitis with uniformly severe strictures. Despite these unfavorable characteristics, endoscopic stricturotomy achieved a technical success rate of 95.2%, comparable to nondiverted patients, with no procedure-related perforation, bleeding, or mortality.

Diversion pouchitis is a common complication in patients with diverted ileal pouches, mainly attributed to the loss of short-chain fatty acids following the exclusion of luminal flow, leading to mucosal inflammation, friability, fibrosis, and possible progressive stricture formation.^4^^,^^16^ Short-chain fatty acids, particularly butyrate, serve as the principal energy source for mucosal epithelial cells, and their absence in the diverted bowel results in mucosal atrophy and chronic inflammation.^17^, ^18^, ^19^ Notably, diversion colitis in patients with underlying IBD is endoscopically and histologically more severe than in non-IBD patients, with inflammation reported in virtually all diverted segments.^5^^,^^17^^,^^20^^,^^21^ When strictures develop in this setting, management is particularly challenging due to the friable, inflamed mucosa and the uniformly severe or pinhole nature of the strictures, as demonstrated in our cohort. Endoscopic stricturotomy has been established as an effective and safe modality for treating fibrotic pouch strictures in nondiverted patients, with technical success rates of 97%–100% and surgery-free survival of 85%–92% in large cohort studies.^5^^,^^10^^,^^11^^,^^22^ However, its application in diverted pouches has been limited to a single case report, in which a patient with UC who had undergone IPAA with diverting loop ileostomy developed a completely sealed pouch outlet with concurrent diversion pouchitis, successfully treated by wire-guided needle-knife stricturotomy.^13^ EBD, though widely used with high short-term success in strictures of nondiverted pouches, has not been systematically reported in patients with diverted pouches.^23^ The tissue in the stricture and adjacent mucosa is friable, even to simple air insufflation during pouchoscopy, is vulnerable to mechanical trauma for EBD. In addition, the radial force of EBD may theoretically disrupt anal sphincters. By incising rather than stretching the tissue, stricturotomy with a circumferential force at the posterior wall of the stricture may provide a more effective, yet safer approach than EBD.

While fecal diversion has been associated with higher rates of anastomotic stricture formation in IPAA patients,^7^^,^^24^ the endoscopic characteristics and treatment outcomes of these strictures have not been specifically studied. Existing studies on the endoscopic management of pouch strictures, whether evaluating balloon dilation, stricturotomy, or surgical strictureplasty, have not stratified treatment outcomes by diversion status.^5^^,^^10^^,^^11^^,^^22^^,^^23^ Our study fills this critical gap by providing the first controlled comparison of endoscopic stricturotomy outcomes between diverted and nondiverted pouch strictures.

Our study adds several novel contributions to the current literature. We demonstrated that diversion pouchitis and stricture formation frequently coexisted, with 75% of diverted patients having concurrent diversion pouchitis. The uniformly severe nature of diverted pouch strictures (100% severe/pinhole) contrasts sharply with the predominantly mild-to-moderate strictures seen in nondiverted pouches, suggesting that the pathophysiology of diversion-associated strictures differs fundamentally from that of strictures in functioning pouches.^3^^,^^4^ The significantly higher PDAI endoscopic subscores in diverted patients further support this distinction. Moreover, the greater postoperative use of small molecule agents (*P* = .046) and a trend toward greater biologics use (75% vs 48%, *P* = .077) in the diverted group suggest that these patients represent a more medically refractory population, which may contribute to the more severe stricture phenotype. Additionally, a numerically higher proportion of diverted patients had CD as the present diagnosis (42% vs 19%), a condition with a known propensity for fibrostenotic complications that may further account for the stricture severity observed in this group. Despite these more challenging stricture characteristics, our data demonstrate that endoscopic stricturotomy is feasible in diverted pouches, with a technical success rate of 95.2% and long-term outcomes comparable to those in nondiverted patients. This technical success rate is reassuring and consistent with the 97%–100% reported in nondiverted populations.^5^^,^^10^^,^^11^ The rate of surgical intervention was comparable between groups (25% vs 21%), and survival did not differ significantly, though the consistently elevated HRs in the diverted group suggest a trend that warrants validation in larger cohorts.

The clinical implications of our findings are noteworthy. Pouchoscopy should be considered in diverted pouch patients even in the absence of stricture-related symptoms, given concerns for occult stricture formation and pouch dysplasia in the chronically inflamed, defunctionalized mucosa. Because obstructive symptoms are unreliable in diverted pouches, often reflecting stomal or neo-distal ileal narrowing rather than the pouch-outlet stricture, endoscopic surveillance rather than symptoms is needed to detect these strictures. Indeed, all diverted patients in our study presented with severe strictures, supporting the role of endoscopic surveillance as recommended by current guidelines.^9^^,^^25^ We therefore recommend annual pouchoscopy for diagnosis, surveillance, and endoscopic therapy in patients with a diverted pouch, even when asymptomatic. The significantly higher use of hemostatic agents in diverted patients (50% vs 14%, *P* = .007) underscores the need for endoscopists to be prepared for hemostasis during stricturotomy in diverted pouches, likely reflecting the friable and inflamed mucosa associated with diversion pouchitis. Endoscopic stricturotomy should be considered a viable therapeutic option for diverted pouch strictures before surgical alternatives such as pouch excision or pouch redo^1^^,^^26^ as successful treatment may ultimately enable ileostomy closure and restoration of intestinal continuity.^6^

An important and underrecognized concern is the risk of infectious complications associated with diverted pouches. The chronically inflamed, friable mucosa and obstructed luminal environment of diverted pouches may predispose to bacterial translocation, particularly during endoscopic intervention. In our cohort, infectious events including a postprocedure pelvic abscess and a suspected diversion-associated liver abscess were observed, consistent with prior case reports of abscess formation in diverted bowel.^27^^,^^28^ Because immunosuppressant use was comparable between groups, the clustering of infectious events in diverted patients likely reflects the chronically inflamed, obstructed luminal environment rather than immunosuppression alone. These findings, while hypothesis-generating given our small sample, highlight the need to consider antibiotic prophylaxis for endoscopic procedures in diverted pouches. Previous guidelines recommend prophylaxis for obstructed luminal systems but do not specifically address diverted pouches.^29^ Notably, this study suggests that prophylactic antibiotics should be considered for all patients with a diverted pouch, highlighting the need for further investigation. Additionally, the management of concurrent diversion pouchitis with appropriate medical therapy, including short-chain fatty acids enemas, mesalamine, or corticosteroids,^30^^,^^31^ may complement endoscopic stricture treatment and optimize outcomes.

This study has several limitations. First, the retrospective, single-center design is subject to inherent biases, including selection and information bias. Moreover, the sample size of diverted patients was small (N = 12), which limits statistical power and precludes multivariable analysis. In addition, the median follow-up was shorter in the diverted group (15 vs 28 months), which may underestimate the long-term recurrence and complication rates. Furthermore, whether pouch strictures existed prior to diversion could not be determined in patients with long-standing diverted pouches, as our data captured only the index endoscopic treatment at our institution. Consequently, the precise interval from diversion to stricture development could not be estimated. Likewise, the lack of a standardized prophylactic antibiotic protocol limits our ability to assess the role of antibiotic prophylaxis in preventing infectious complications. Additionally, given the retrospective design, adverse-event ascertainment was limited to events documented in available medical records and may underestimate the true incidence, particularly for events evaluated or managed at outside institutions. Finally, these results were achieved at a tertiary referral center by a single endoscopist with extensive experience in interventional IBD endoscopy. Endoscopic stricturotomy, particularly needle-knife stricturotomy of severe or pinhole strictures, is a technically demanding procedure that may not be readily available outside specialized referral centers; our findings may therefore not be generalizable to community-based practice, and referral to experienced centers should be considered for complex diverted pouch strictures. Despite these limitations, this study provides the first controlled data on a clinically important yet understudied topic and establishes a foundation for future prospective investigation.

## Conclusion

The vast majority of patients with strictures in the diverted pouch had concurrent diversion pouchitis. The strictures in the diverted pouch turned to be more severe and longer than those in the nondiverted pouch. Endoscopic stricturotomy was feasible in treating strictures in the diverted pouch with a comparable outcome to the strictures in the nondiverted pouch. Clinicians should be aware of the potential risk of infectious complications in diverted pouches, and antibiotic prophylaxis warrants consideration.
